# Relation between Psychological Restorativeness and Lifestyle, Quality of Life, Resilience, and Stress-Coping in Forest Settings

**DOI:** 10.3390/ijerph16081456

**Published:** 2019-04-24

**Authors:** Norimasa Takayama, Takeshi Morikawa, Ernest Bielinis

**Affiliations:** 1 Forestry and Forest Products Research Institute, Forest Research and Management Organization, Matsunosato, Tsukuba, Ibaraki 305-8687, Japan; tmori@ffpri.affrc.go.jp; 2 Department of Forestry and Forest Ecology, Faculty of Environmental Management and Agriculture, University of Warmia and Mazury, Pl. Łódzki 2, 10-727 Olsztyn, Poland; ernest.bielinis@uwm.edu.pl

**Keywords:** lifestyle, mood states, perceived restorativeness scale, positive and negative affect schedule, quality of life, resilience, restorative outcome scale, Shinrin-yoku, stress coping, subjective vitality scale

## Abstract

Previous research has mainly dealt with the physiological and psychological restorative effects of the forest environment. However, comparatively few studies have focused on how the traits and attributes of individuals (individual traits) affect the restorative effects of the forest environment. In this study, we examined the relationships between the psychological restorative effects offered by perceived restorativeness of outdoor settings and the individual traits. Then, we investigated the relationships between the restorative indicators that are useful in examining the restorative properties (i.e., the Perceived Restorativeness Scale (PRS); seven indicators in total), the psychological restorative effect (Profile of Mood States (POMS), Restorative Outcome Scale (ROS), positive and negative affect schedule (PANAS), and Subjective Vitality Scale (SVS); 10 indicators in total), and the individual trait indicators that could be used to investigate individual traits (Development of Health and Life Habit Inventory for lifestyle, Lazarus-type Stress Coping Inventory for stress coping, World Health Organization Quality of Life Assessment 26 for quality of life (QOL), and Sukemune-Hiew Resilience test for resilience; 28 indicators in total) in forest and urban settings. Respondents consisted of 46 male students in their twenties. A short-term experiment was conducted using the same method in both environmental settings. We then analyzed the intrinsic restorative properties and the restorative effects of the settings and referred to prior research to determine the restorative effects. Furthermore, we analyzed the relationship between the restorative indicators and the individual trait indicators by correlation analysis and multiple regression (step-wise) analysis. These new findings were obtained: (1) the forest setting was a restorative environment with a higher restorative effect than the urban setting; (2) although the forest setting had a higher restorative effect than the urban setting, and the influence of individual traits was small; (3) in the forest setting, the relationship between the restorative indicators and individual traits indicators were arranged; (4) distancing (Stress coping), psychological health (QOL), and satisfaction with living environment (QOL) were likely important indicators that are related to the restorative effects in the forest setting.

## 1. Introduction

With urban life in the modern world, people experience many stressors, which increasingly drive them to seek some form of stress reduction. In the recently urbanized environments and societies, chronic and intense stress, and a deficiency in restoration from stress are well recognized as increasing problems that have long-term negative effects on health [1,2,3]. Poor stress management is related to psychological issues, such as depression, panic disorder, and burnout syndrome; as well as physical problems, such as neurological, cardiovascular, immunological, and gastroenterological diseases [4,5].

Due to the social problems caused by urban stress, there is an increasing focus on the potentially restorative effects of the natural environment. Lots of evidence on the positive effect of the natural environment on health and happiness has already been published. As a principle, the physiologist Miyazaki advocated using Miyazaki’s Nature Therapy Theory to improve health, which was based on an evolutionary anthropological perspective [6]. For example, many studies have considered the physiological effects. One such study compared the physiological effects of walking in and viewing a forest versus a control (i.e., mainly urban) area. It showed that physiological parameters, such as the pulse rate and diastolic blood pressure, were lower for the forest than the urban [7]. Some other studies contributed by having walking and viewing in reverse order [8,9,10]. Haluza et al. [11] investigated the restorative effect of the physical environment. According to the authors, numerous works concluded that the natural environment is better at reducing stress than the urban environments (where most people live their daily lives) by promoting physical well-being (i.e., lower blood pressure, lower muscle tension, higher skin conductance, higher brain activity, etc. [12,13,14,15,16,17]). Kühn et al. [18] investigated the associations between geographical properties and brain functioning in terms of brain activity. They found that living near a forest was associated with a healthier amygdala, and suggested that people who lived near the forest would be happier than those who lived away from it.

The Attention Restoration Theory (ART) proposed by Kaplan and Kaplan (1989) [19] help elucidate why natural and forest environments are beneficial to psychological health. Roe and Aspinall [20] examined the psychological recovery effect on psychiatric patients and reported that taking walks in a rural area benefited the affective restoration and cognitive restoration, particularly for people with poor mental health. In addition, some studies on environmental designs made use of these beneficial effects. Ulrich [21] suggested that in hospitals, surgical patients assigned to rooms with windows facing natural scenery (including trees) had shorter post-operative hospital stays, had fewer negative comments from the nurses, and required fewer potent analgesics than those assigned to similar rooms but with windows facing a brick wall. Besides, Velarde et al. [22] indicated that natural environments, including urban parks and several types of forests, were strongly associated with higher energy levels and had other positive health effects in contrast to the urban environment. Recently, some studies reported that the Multiple Mood Scale-Short Form (MMS) scores were higher for friendliness and well-being on days spent walking in the forest compared with the control days. This was especially true among individuals who felt chronic mental stress [23,24]. The MMS score for depression and State-Trait Anxiety Inventory (STAI) [25] score were also lower on the forest days [26]. The psychological responses were also measured by the profile of mood states (POMS) [7,9,27], Zuckerman inventory of personal reactions (ZIPPERS) [28], restorative outcome scales (ROS) [29,30,31], subjective vitality scale (SVS) [32], and positive and negative affect schedule (PANAS) [33,34]. All indicators showed that the forest setting has more positive psychological restorative effects than the urban setting.

It has been previously mentioned that there may be influences from inter-individual differences on the restorative effect of forest bathing. For example, using on-site experiments, Koyama et al. [35] found that even where users experienced the same forest environment, there were individual differences in how they experienced the environment and the restorative effect obtained. There is a possibility that the health-restoring effects of the forest do not equally benefit all users. This has not yet been explored in specific details; however, Takayama et al. [36] indicated that individual trait differences can act as filters when interpreting the forest setting. The differences were first generated from how the user selects the primary stimulus (e.g., visual) from the forest setting, then also in the quality and degree of the restorative effects obtained from the time spent in the forest. Previous studies that investigated the effects of individual traits on the restorative effects of forests only considered values on personality [36,37,38], self-efficacy [36,37,39], living environment [36,37], preference and experience [36,37]. Because the factors that people find to be healing differs among individuals, and how the mind and body are restored is a very personal phenomenon. As a result, the possibility that individual traits can influence the restorative effects could not be ruled out. Also, Kaltenborn et al. [40] mentioned that it is necessary to design environments with consideration for the diversity of individuals and specific groups (e.g., culture and/or preference, and so on).

We also agree that it is desirable to provide the most effective choice in consideration of the user’s traits, even in future studies aiming to further demonstrate the health and recreational benefits of the forest environment. Therefore, in this study, to clarify whether individual traits affect the restorative effects of the forest setting and to elucidate which indicators of individual traits affect the restorative effects, we attempt to identify the properties of the forest setting that are considered to have high restorative effects using prior research. Then, we also discuss the relationships between the representative individual traits and the restorative effects of the forest setting.

## 2. Materials and Methods

### 2.1. Research Sites

**Figure 1:**
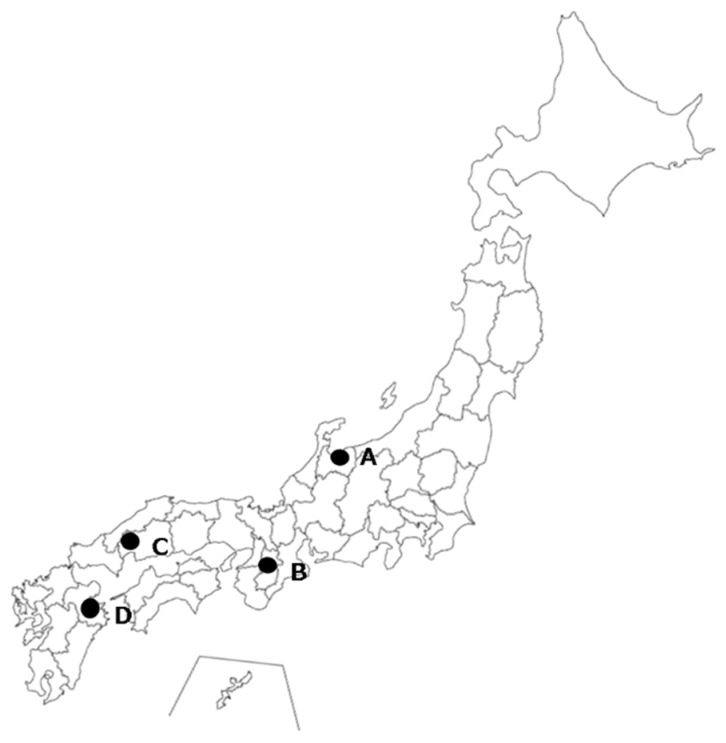
Location of the four study municipalities in Japan (**A**–**D**).

The experiments were carried out in four municipalities. The characteristics of each municipality, forest, and urban sites are presented in Table 1. Four forest sites and four urban sites were selected. The urban sites were located near the forest sites. Specific details on the locations of the sites are presented in Figure 1. The urban setting was used as control. The sites were chosen from the area where the respondents reside, as the area should be familiar to the respondents. Our method of site selection for the urban setting was commonly used in other similar studies [7,8,9,10,17,41,42,43]. Using this site selection method should make our results comparable with those of prior studies. The forested sites of Kamiichi (A) and Yoshino (B) are artificial plantations consisting mainly of coniferous trees (i.e., Japanese cedar). Akiota (C) and Oita (D) consist of deciduous forests (i.e., Japanese oak, Sawtooth oak, and others). These forest areas were well-managed, had high levels of lighting, and were relatively flat. The urban sites were located mainly around the downtown major traffic routes, or near the main train station in each municipality. The total experimental period, average number and age of respondents, average temperature and humidity values, and weather conditions throughout the experiment in each site of the four municipalities are presented in Table 2.

### 2.2. Respondents

We controlled for the variation between respondents to avoid the influence of differences in attributes, such as age or gender, by selecting 46 young male undergraduate and graduate university students as respondents (Table 2). We asked the municipalities’ staff at each research site to recruit respondents from the universities nearby. The respondents were not familiar with the study and the underlying theory, as they belonged to a variety of other faculties. By excluding students from the related academic field (e.g., environmental management), academic field-specific bias was avoided. The students were hired by the authors as a research subject over the experimental period. None of the respondents had reported any history of physical or psychiatric disorders. The experiments were conducted in accordance with the Declaration of Helsinki. The research protocol was approved by the Ethics Committee of the Forestry and Forest Products Research Institute in Japan (22FFPRI-1884). All respondents were fully informed of the aims and procedures of this experiment. Their informed consent was obtained prior to the experiment.

### 2.3. Experimental Schedule

**Figure 2:**
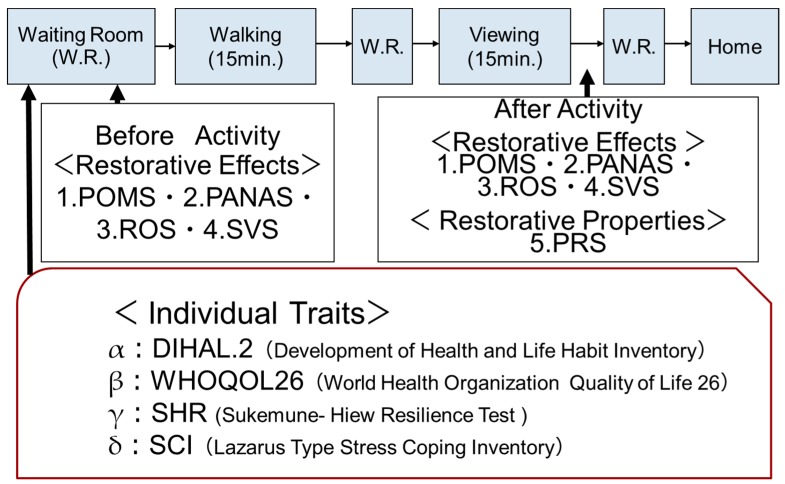
Experimental protocol of the study.

The experiment was conducted over a period of 2 days. The respondents were gathered at a meeting point on the morning of the first day. They were then taken to the meeting room for an orientation where they received an explanation of the experimental procedures. They were required to sign a consent form. After the orientation, the respondents were randomly divided into two groups, each with five to six respondents. On the first day, the respondents in one of the groups were taken to the forest sites, while the respondents in another group were taken to the urban sites. On the second day, the same respondents were taken to the other opposite setting (i.e., forest group into the urban site, and vice versa) to avoid order bias. In a waiting room, we administered four questionnaires, α, β, γ, and δ (Figure 2). These were prepared for each site to investigate the respondents’ individual traits (first day only). After which, we requested answers to the four questionnaires (1 to 4; both days) to investigate the baseline before spending time in each setting. After submitting the questionnaires, the respondents were instructed to leave the waiting room and walk a course independently for about 15 minutes for each setting (i.e., walking). Upon returning to the waiting room, they were given a short break and were instructed to sit alone on a chair placed in a representative location within each setting for 15 minutes (i.e., viewing). After viewing, to investigate the restorative effect and restorative properties of each setting, we sat the respondents down and again asked them to complete the same questionnaire (1 to 4), plus questionnaire 5.

### 2.4. Measurement

In this study, we tested the hypothesis that the traits of each individual had an influence on the restorative effects received from both settings. As a part of investigating the restorative effects of the forest and the urban setting, it is also important to investigate the restorative properties via the perception of the respondents. Thus, we compared the perception of the respondents with the results obtained from the restorative properties. We measured the data based on three categories: the restorative property of environment (i.e., restorative properties), the restorative effect from the environment (i.e., restorative effects), and the personality traits (i.e., individual traits).

#### 2.4.1. Measurement and Comparison of the Restorative Properties

We used the Perceived Restorativeness Scale (PRS) and requested all respondents to complete the questionnaire to investigate the restorative properties of the forest and urban settings. The PRS was developed by Hartig [44] and was modified based on Kaplan and Kaplan’s ART [19]. The Japanese version of the PRS was directly translated by Shibata et al. [45]. It is comprised of 26 items measured based on 11-point Likert scales (Table 3). The theory by Kaplan et al. [19] has four elements of restorative properties: “Being away,” ”Fascination,” ”Extent,” and ”Compatibility.” However, the PRS consists of seven elements including the different from ART’s elements. In particular, the “Extent” element was further divided into "Coherence” and "Scope” elements. The PRS additionally measures "Familiarity” and "Preference.” The extent to which a particular environment restores mental alertness can be measured by including the elements “Being away,” “Fascination,” “Coherence,” “Scope,” “Compatibility,” “Familiarity,” and “Preference.”

#### 2.4.2. Measurement and Comparison of the Restorative Effects

The POMS is a well-established, analytically-derived factor-based measure of psychological distress. Its validity and reliability are well documented [46]. We used the Japanese version of POMS (covering 65 queries) [47] and its raw data for statistical analysis. The PANAS [33,34] measures the positive and the negative affect through the use of 20 items (10 each for positive and negative affect). We used the Japanese version of the PANAS (covering 16 items; 8 of positive and negative affect each) [48]. The ROS can be used to investigate the restorative emotional and cognitive outcomes in a given environment using the six items. The ROS is based on previous measurements and findings regarding restorative outcomes [29,30,31]. We used the Japanese version of the ROS (covering all six items) [49]. There are currently two versions of the SVS. One of the versions assesses the enduring traits of individuals. The scale is positively related to self-actualization and self-esteem and is negatively related to depression and anxiety. The other version assesses the state of subjective vitality rather than the enduring aspect. The versions have four items in common and seven items that are different. The reliability and validity of the SVS were confirmed in previous studies [32,50]. In this experiment, we used the four common items to generate a Japanese version of the SVS questionnaire [51]. These four questionnaires have already been used previously in Takayama et al. [41] and Bielinis et al. [42]; therefore, the psychological restorative effect is verifiable from a composite viewpoint (Table 3).

#### 2.4.3. Measurement and Comparison of Individual Traits

In some cases, it was difficult to distinguish between traits and attributes from the indicators of the individual traits consisting of the four items. Here it was not critical to distinguish between these in a strict sense; therefore, we interpreted the meanings of the individual traits more broadly. Even if the original meaning of the term contained elements of the attribute, we defined the term in relation to the individual traits. Our investigation focused on four major psychological aspects: health and lifestyle habits, quality of life, stress coping, and resilience (Table 3). The usefulness of the four aspects was outlined by Li et al. [13,43], who discussed the potential differences in the effects of forest bathing depending on the lifestyle habits of individuals. Takayama et al. [52,53] also investigated the effects of forest bathing on Quality of Life (QOL) [52], resilience, and stress coping [53]. We then referred these to the individual traits considered important. To assess the health and lifestyle habits, we used the Japanese version of the DIHAL.2 (Development of Health and Life Habit Inventory) that was developed by Tokunaga [54]. The questionnaire was developed to diagnose problems in health and lifestyles and to provide a reference point for health guidance. It comprises each of the five indicators: health, exercise, meal, rest, and lifestyle habits. QOL indicates the quality of the activities in a person’s life and the quality of life from the perspective of society. It is based on observing how a person can live and be independent, or find happiness. In the present study, the Japanese version of the WHOQOL 26 (World Health Organization Quality of Life Assessment 26) [55] was used to assess the QOL of the respondents. WHOQOL 26 uses five indicators: physical health, psychological health, social relationships, the quality of an environment, and the result of a comprehensive evaluation (Total). Stress coping refers to the process of perceiving stress and adapting to it [56]. To investigate the respondents’ stress coping abilities, we used the Lazarus-type stress coping inventory (SCI) questionnaire. The SCI measures the respondents’ stress coping abilities using 10 indicators [57]. The test can allocate two strategies and eight approach types. In contrast to stress coping, resilience refers to an individual’s ability to adapt one’s life activities in the wake of a disadvantageous environment, which could involve family, relationships, health problems, and social risk advantage in the workplace [58]. The Sukemune-Hiew Resilience test (SHR) was used to investigate resilience. The SHR includes the eight indicators allocated to Part 1 and Part 2 of the questionnaire. In Part 1, the questionnaire measures resilience using four factors. Part 2 is concerned with the extrinsic-oriented attitude (represented by action) and the intrinsic-oriented attitude (represented by thinking). We can investigate the four patterns: I: Active (extrinsic)–Active (intrinsic), II: Passive (extrinsic)–Active (intrinsic), III: Active (extrinsic)–Passive (intrinsic), and IV: Passive (extrinsic)–Passive (intrinsic), which are divided into active and passive combinations [59].

### 2.5. Analysis

After categorizing the survey results of the PRS, we compared the restorative effects between the forest and urban settings for each of the seven indicators mentioned above. We used paired t-test and Bonferroni’s correction to avoid Type-I errors (Table 4). The results for individual traits for each indicator were tabulated (Table 5). To compare the restorative effects, we reorganized the part of data used in Takayama et al. [41] and used a paired t-test for each indicator. We compared the scores obtained from each setting before and after staying (Table 6), and the scores from before and after time spent in each setting (Table 7). Subsequently, to investigate the basic trends in the relationship between restorative effects and individual traits, correlation analysis was performed in each of the urban and forest settings. Although correlation analysis could occasionally reveal that the relationship was not only potentially influenced by other factors (possibility of multiple collinearities) but also consisted of type-I; error (statistical reliability), the authors considered it an effective method for grasping the general relationship between the two factors, while acknowledging its limitations. Correlation analyses (uncorrelated tests) were performed for all indicators of individual traits and the restorative effects of each environmental setting to elucidate the overall trends (Table 8 and Table 9). Also, to eliminate the influence of other factors as much as possible and to investigate which restorative effects and individual traits were strongly related, we referred to prior research and investigated an effective method for exploring the relationships among multiple factors [60,61]. Therefore, to investigate the relationship from a different perspective using correlation analysis, we attempted to use multiple regression analysis (step-wise, forward selection) with indicators of restorative effects as dependent variables and indicators of individual traits as independent variables (Table 10). The statistical analyses were carried out in IBM SPSS Statistics 25 (IBM).

## 3. Results

### 3.1. Comparison of Restorative Properties between Forest and Urban Sites

In the comparison between forest and urban sites using the PRS score, significant differences were found in all indicators except for “Fascination” and “Coherence.” “Being away,” “Scope,” “Compatibility” and “Preference” were significantly higher in the forest sites, and only "Familiarity" was significantly higher in the urban sites. As for “Fascination” and “Coherence,” there was little difference in the scores among the two different sites and no significant difference between the two sites could be confirmed (Table 4).

### 3.2. Restorative Effects and Individual Traits

Table 5 summarizes the average values and the standard deviations of the 28 indicators used as measures of individual traits. The results of the restorative effect between the forest and urban site are shown in Table 6. Before spending time in the respective settings, there were no significant differences among sites in any of the restorative effect indicators in terms of the participants’ psychological state. In contrast, after spending time in the respective settings, there were significant differences among sites for “T-A, ” “V, ” “F, ” “C” (POMS), “Negative affect” (PANAS), “ROS” and “SVS.” A positive psychological restorative effect was observed across sites for the forest setting. 

Table 7 shows the results from the psychological measurements before and after staying at each site. In the forest site, “T-A” decreased significantly after staying. In contrast, “SVS” increased after staying. Meanwhile, “SVS” decreased significantly in the urban site, and “T-A” (POMS), “Negative affect,” and “Positive affect” increased significantly. Therefore, the results from the forest and urban setting were very different.

### 3.3. The Relationship between Individual Traits and Restorative Effects

#### 3.3.1. Correlation Analysis

To investigate the overall trend of the relationship between the restorative effects and individual traits for each environmental setting, correlation analyses were performed for all indicators (Table 8 and Table 9). The restorative effect index consisted of 10 indicators. Net values were obtained by subtracting the restorative effect obtained before from the values obtained after spending time in the site. The individual traits index, which consists of 28 indicators, was used as the individual traits indicator. In the forest setting, 7 out of 280 correlations were significant (Table 8). In the urban setting, 17 of 280 correlations were significant (Table 9).

#### 3.3.2. Multiple Regression Analysis (Step-Wise)

We conducted multiple regression analyses (step-wise, forward selection) to investigate further how the indicators of individual traits influenced the restorative effect. In the forest setting, there were significant relationships in 5 out of 280 cases. On the other hand, in the urban setting 12 out of 280 relationships were significant. Table 10 presents the results from multiple regression analysis and provides information on the direction of the relationships.

## 4. Discussion

### 4.1. The Restorative Properties of Environmental Settings and Restorative Effects

We assessed the restorative properties of the forest and urban settings and the psychological restorative effects. First, the scores related to daily life, Being away, Scope and Compatibility were statistically higher in the forest setting than in the urban setting (Table 4). This result implies that the forest setting has higher restorative properties than the urban setting. The ART of Kaplan and Kaplan [19] indicates that an environment has four functions that can exert beneficial restorative effects: (1) Being away (feeling refreshed away from everyday occurrences), (2) Fascination (that which enthralls people and attracts interest), (3) Extent (which infuses feelings of spatial expanse; divided into Coherence and Scope in the PRS), and (4) Compatibility (which makes the environment feel suitable). From our results, an appropriately managed forest setting could be an excellent restorative environment in comparison with the urban setting, the latter of which is where the majority of people live. On the other hand, in the comparison of the restorative properties to the restorative effects, there was no significant difference between the forest and urban settings for participants in all indicators before spending time in each of the respective settings (Table 6). However, after the respondents have spent time in the forest, they attained a significantly more positive psychological state than after spending time in the urban setting (Table 6), and even before staying at the forest setting (Table 7).

Overall the four forest sites are restorative environments with higher restorative effects in contrast to the four urban sites. The higher psychologically restorative effect may be because of the highly restorative properties of the forest acting as a stimulus to the respondents.

### 4.2. The Restorative Effects and Individual Traits in the Forest and Urban Settings

#### 4.2.1. Forest Setting

There was a significant correlation between the composite index of the 10 restorative effects and the index of 28 individual traits. More specifically, in the forest setting there were significant correlations between Vigor (POMS) and Distancing (Stress coping), Fatigue (POMS) and Environment (QOL), Confusion (POMS) and Meal (Health and life habit), Confusion (POMS) and Environment (QOL), SVS and Distancing (Stress coping), and Emotion-focused (Stress coping) and Psychological health (QOL; Table 8). In other words, stress coping ability of the “Distancing type” (i.e., thinking that problems are not related to oneself and trying to forget problems and suffering) was inversely correlated with the feeling of liveliness and energy after spending time in the forest. The environmental area of QOL (i.e., a degree of satisfaction with one’s living environment on a daily basis) was inversely correlated with fatigue and confusion after spending time in the forest. Since both Vigor (POMS) and SVS are restorative indicators of the psychological state, people who have a relatively low ability to cope with the distancing type of stress (i.e., those who confront themselves without escaping from problems) are more likely to increase in psychological vigor from spending time in the forest setting. 

Furthermore, the distancing type of stress coping had a negative relationship with Vigor (POMS; −0.338) and SVS (−0.295). The environmental area of QOL had a negative relationship with Fatigue (POMS; −0.560) and Confusion (POMS; −0.335; Table 10). This suggests that people with distancing characteristics may have a lower psychological state of vigor from staying in a forest setting. People who are satisfied with their daily living environment may also have less fatigue and confusion. These results are reflected in the results of the correlation analyses. Thus, psychological health has a negative relationship with SVS (−0.325). In addition, people dissatisfied with their psychological health aspect of their QOL tended to experience greater effects on subjective vitality. 

These results suggest that factors related to lifestyle and resilience do not necessarily affect the effect of staying in a forest setting. Li et al. [13,43] indicated that forest staying is effective for treating lifestyle diseases and Takayama et al. [53] suggested that psychological resilience is improved by staying in a forest. However, if we considered psychological resilience as factors of individual traits, there was a possibility that it did not necessarily affect the effect of a single and short-term forest staying. Some indicators of stress coping and QOL were considered as factors influencing the effect of short-term forest staying. Takayama [36] analyzed the relationship between the big five factors of personality and the restorative effects by forest staying. He hypothesized that only a limited number of factors would have an influence on the effect of forest staying. Our results provide support for their hypothesis.

#### 4.2.2. Urban Setting

The equivalent analysis was carried out with the urban setting as the control (Table 9). There were 17 significant correlations. Some significant correlations were obtained compared with the forest setting (7 cases). In particular, among the individual traits, the indices for stress coping (10 indicators) was significantly correlated with many restorative effect indicators. For example, the self-control, escape, distancing, and emotion-focused types were significantly negatively correlated with Vigor (POMS). In addition, for the passive (extrinsic)–active (intrinsic) type (Resilience), the environmental area (QOL) was significantly correlated with positive emotions (PANAS). Thus, the directions of correlation between individual traits and the restorative indicator were variable. 

According to the results from the multiple regression analyses (Table 10), there were 12 significant relationships between individual traits and the restorative indicator. Taking negative emotion (PANAS) as an example, people who are of the self-control type (i.e., stress coping: take countermeasures to deal with stress by self-control; 0.597), and not those who are the problem-focused type (i.e., stress coping: tries to solve problems face to face; −0.413) and the passive (extrinsic)–active (intrinsic) type (i.e., resilience: people whose behavior is active but the way of thinking is passive; 0.285), tended to have negative emotions after spending time in the urban environment. Our results suggest that by staying in the urban setting, people who are of the self-control type and active (extrinsic)–passive (intrinsic) type (i.e., resilience: behavior is passive, but the way of thinking is negative) may experience reduced psychological restoration (ROS). As described above, our results provided details on how individual traits have a significant influence on the restorative effect of spending time in urban areas.

In the urban setting, there were significant influences from the three categories (i.e., lifestyle, stress coping, resilience). In addition, we found that the psychological state was affected by more individual traits from staying in the urban setting than in the forest setting. The urban setting was not a restorative environment compared to the forest, and a restorative effect was not expected either. However, in the urban setting, as a strong stimulus was scattered (in contrast to the forest setting), it was necessary for each person to pay more attention to sufficiently adapt to their environment. For this reason, we considered that many more individual traits were related to the stay in the urban setting rather than in the forest setting.

#### 4.2.3. Comprehensive Discussion

When the results for the forest and urban settings were compared, both correlation and multiple regression analyses revealed many significant relationships in the urban setting (17 for the correlation analysis and 12 for the multiple regression analysis). On the other hand, fewer significant relationships were revealed in the forest setting (7 for the correlation analysis and 5 for the multiple regression analysis). As previously discussed, a forest setting has a higher restorative effect than an urban setting (Table 6 and Table 7) because of its higher restorative properties (Table 4), the same as the previous studies [7,8,9,10,17,42,43]. Thus, if the same amount of time was spent in both settings, a higher restorative effect should be obtained in the forest setting. Considering that a forest setting has a higher restorative function, the differences in the number of significant relationships that were revealed in this study implies that it is also affected by individual traits, and not simply due to the effect of the environment alone. In other words, the restorative effect obtained in an urban setting tends to be strongly influenced by the individual traits of the respondents. In the forest setting, it is thought that there was a stable restorative effect with little influence from the variation in individual traits. Although there were only a few significant relationships in the forest setting, there were some significant relationships between individual traits and the restorative effect of the environment. How we relate to the environment and feel about the environment varies among individuals. Therefore, as we have revealed, it is important for the forest management and planning, as well as for forest experience programs to consider the knowledge from research on the different effects that spending time in a forest setting have on people with different individual traits [26,36]. For example, if we would like to improve the psychological state of the vigor of a participants’ gpoup, we should investigate their stress coping mechanisms in advance and then expose the participants to different distancing type and non-distancing type of environments and programs [53]. We will then be able to provide more effective services. From these findings, it was clear that individual traits (such as the distancing type of stress coping) and the psychological and environmental areas of QOL are related to restorative effects. These have not been dealt with previously. Regarding the effect of individual traits, which not been given much attention in previous studies, on the planning of forest use and management, we believe that more concrete knowledge could be acquired that will enable more effective forest planning, with consideration of individual traits. Takayama [36] showed that during a walk for a short-term in a forest setting alone, the restorative effect of forest staying was high for people who have high neuroticism, and people who have high extroversion had lower effectiveness. Furthermore, it is important to develop a forest bathing program suited for each individual trait type. Since we found that three factors, namely (1) distance from the stressor to protect oneself from stress, (2) satisfaction with daily living environment, and (3) psychological health, were related to the effect of single and short-term forest staying, the results from this study can be considered to use in the arrangement of a new and diversified program.

## 5. Conclusions

In the present study, we investigated and analyzed the relationships between individual traits (i.e., health and lifestyle, stress coping ability, resilience, and QOL) and the restorative effects from a short stay in a forest setting. The following findings were obtained.

The forest setting is a restorative environment with a higher restorative effect than the urban setting.

Although the forest setting had a higher restorative effect than the urban setting, the influence of differences in individual traits was minimal.

The relationships between restorative indicators of the forest environment and individual traits indicators were elucidated.

Distancing (stress coping), psychological health (QOL), and satisfaction with the living environment (QOL) were potentially important indicators related to the restorative effects in the forest setting.

The above results were obtained even though the time the respondents spent in the forest settings was relatively short. Therefore, regardless of individual traits, people can experience a psychological restorative effect by simply spending time in a forest setting for a relatively short period (approximately 30 minutes to 1 hour). Several studies explored the effects of individual differences on the effect of staying in the forest setting, such as Takayama [36] and Takayama et al. [37]; however, none used the indicators of individual trait types explored herein, where we analyzed the influence of the forest setting compared with the urban setting (control). Therefore, the present study is exploratory. There were numerous indicators of individual traits that could not be accounted for in the present study. Also, the number of respondents was limited due to the use of direct recruitment of students on-site.

Furthermore, we chose young men in their 20s as respondents to control for the respondent’s attributes. The effects of gender and age would require further exploration in the future. The relationships between cognitive differences in individual respondents (i.e., restorative properties) and differences in restorative effects between forest and urban settings (i.e., restorative effects), or the relationship between cognitive differences and individual traits have not been studied previously. Exploring such dynamics is important for establishing the overall relationships among individual traits and restorativeness, which should be elucidated further in future studies. Although a well-managed forest environment is highly beneficial to users regardless of individual differences, further research could focus on developing optimal forest management and forest experience programs, while taking into account differences among individual users.

The present study had some limitations. First, with regard to correlation and multiple regression analysis, it may be necessary to consider the potential of the presence of type-I errors. Therefore, readers should be careful when they consider the results of the present study. In addition, the urban setting was used as a control and compared with the forest setting, referring to many previous studies [7,8,9,10,12,13,17,27,35,36,37,39,41,42,43]. However, the results could vary depending on a researcher’s perspective on what should be applied as a control. We would like to see the progress research in this field in the future.

## Figures and Tables

**Table 1:** Summary of the locations of the four municipalities in this study.

Symbol	A	B	C	D
Municipality	Toyama Prefecture	Nara Prefecture	Hiroshima Prefecture	Oita Prefecture
	Kamiichi Town	Yoshino Town	Akiota Town	Oita city
Forest Site	Temple Pillar Approach in Tateyama Mountain	Trailhead at Yoshino Mountain	West Trail in Shin’nyuzan	Forest Road in Oita Prefectural Forest
Urban Site (Control)	Road before City Center	Road before Kintetsu Department Store	Road before Hiroshima Prefectural Government Building	Road before Oita Bank, Ltd.

**Table 2:** Summary of the number of respondents and weather conditions at the four study municipalities (A–D).

Symbol	Experimental Period	Number of Respondents	Age of Respondents	Weather Forest/Control	Temperature (℃) Forest/Control (Average ± S.D.)	Humidity (%) Forest/Control (Average ± S.D.)
A	September 6–7, 2011	11	21.4 ± 1.3	Fine/Fine	25.2 ± 1.49/27.5 ± 0.89	52.0 ± 8.46/41.5 ± 2.83
B	August 3–4, 2011	12	21.2 ± 0.8	Fine/Fine	28.4 ± 2.42/34.5 ± 2.80	64.9 ± 12.9/42.6 ± 8.46
C	August 8–9, 2011	12	20.8 ± 1.5	Fine/Fine	26.6 ± 1.29/34.6 ± 1.44	78.0 ± 6.72/56.6 ± 4.26
D	September 13–14, 2011	11	21.1 ± 1.4	Fine/Fine	28.0 ± 1.80/31.8 ± 0.88	63.2 ± 7.09/59.1 ± 2.52

Temperature and humidity were measured every 10 minutes from 9:00 to 16:00 during the experimental period. There were a total of 42 measurements for each parameter.

**Table 3:** Outline of the questionnaires used in the study.

Name and Abbreviation of Questionnaire	Target of Measurement	Outline of Questionnaire	Before Experiment (B.E.)	After Experiment (A.E.)
Perceived Restorativeness Scale (PRS) *Restorative properties*	Evaluation of restorative properties	7 indicators; Being away, Fascination, Coherence, Extent, Compatibility, Familiarity, Preference	-	○
Profile of Mood States (POMS) *Mood states*	Evaluation of restorative effects	6 indicators; T-A: tension-anxiety, D-D: depression-dejection, A-H: anger-hostility, V: vigor, F: fatigue, C: confusion	○	○
Positive and Negative Affect Schedule (PNANS) *Emotional affects*		2 indicators; Positive affect, Negative affect	○	○
Restorative Outcome Scale (ROS) *Restorativeness*		1 indicator; Subjective restorativeness	○	○
Subjective Vitality Scale (SVS) *Vitality*		1 indicator; Subjective vitality	○	○
Development of Health and Life Habit Inventory (DIHAL.2) *Health and life habit*	Evaluation of individual traits	5 indicators; Health, Exercise, Meal, Rest, Lifestyle habits (Exercise + Meal + Rest)	○	-
WHO Quality of Life 26 (WHOQOL26) *QOL*		5 indicators; Physical health, Psychological health, Social relations, Environment, Total	○	-
Sukemune-Hiew Resilience Test (SHR) *Resilience*		8 indicators; Social support, Self-efficacy, Sociality, Total amount, I: Active (extrinsic)–Active (intrinsic), II: Passive (extrinsic)–Active (intrinsic), III: Active (extrinsic)–Passive (intrinsic), IV: Passive (extrinsic)–Passive (intrinsic)	○	-
Lazarus Type Stress Coping Inventory (SCI) *Stress coping*		10 indicators; Problem-focused coping, Emotion-focused coping, Planful problem solving, Confrontive coping, Seeking social support, Accepting responsibility, Self-controlling, Escape-avoidance, Distancing, Positive reappraisal	○	-

○: Measured point; -: Not measured.

**Table 4:** Results from the comparison of the restorative properties between the forest and control sites (n = 46).

	Setting	Being away	Fascination	Coherence	Scope	Compatibility	Familiarity	Preference
*Ave.*	Forest	34.5	32	22.4	27.5	28.2	3.4	10.9
	Urban (Control)	20.8	27.1	21.2	18.8	23.5	5.4	7.7
*S.D.*	Forest	12.5	9.8	7	9.2	8	2.9	4.7
	Urban (Control)	13.6	10.3	8.7	8.6	6.6	2.7	4.1
*p value*		0.000	0.033	0.101	0.000	0.003	0.001	0.001
*Significance*		**	-	-	**	*	**	**
*effect size: r*		0.568	0.335	0.204	0.610	0.441	0.487	0.519
*statistical power: β*		0.975	0.910	0.148	0.993	0.922	0.853	0.952

Ave. = Average; S.D. = Standard Deviation; *p* values from paired t-test (After applying Bonferroni’s correction). Paired t-test significance levels: ** *p* < 0.00143, * *p* < 0.00714, - *p* > 0.00714.

**Table 5:** Summary results from different questionnaires for assessing individual traits (n = 46).

**Health and Life Habit**					
	*Health*	*Exercise*	*Meal*	*Rest*	*Life Habit*
*Ave.*	41.4	28.6	39.1	42.9	110.6
*S.D.*	6.49	5.86	8.17	7.56	17.52
**Resilience**					
	*Social support*	*Self-efficacy*	*Sociality*	*Total*	
*Ave.*	47.2	34.8	16.6	98.7	
*S.D.*	7.92	6.88	3.97	14.99	
	Active (extrinsic)-Active (intrinsic)	Passive (extrinsic)-Active (intrinsic)	Active (extrinsic)-Passive (intrinsic)	Passive (extrinsic)-Passive (intrinsic)	
Ave.	3.2	2.6	1.4	0.8	
S.D.	1.78	1.34	1.19	1.06	
**Stress Coping**					
	*Planful*	*Confront*	*Seeking social support*	*Accepting responsibility*	*Self-control*
*Ave.*	8.2	6.7	4.7	8.6	7.3
*S.D.*	3.94	2.90	3.48	3.86	3.16
	*Escape*	*Distancing*	*Positive reappraisal*	*Problem-focused*	*Emotion-focused*
*Ave.*	5.7	6.7	8.4	30.0	26.2
*S.D.*	2.60	3.25	4.12	12.55	8.73
**Quality of Life**					
	*Physical health*	*Psychological health*	*Social relationships*	*Environment*	*Total*
*Ave.*	25.1	20.2	10.4	26.3	6.4
*S.D.*	3.69	3.85	2.31	4.06	1.58

**Table 6:** The results of the comparison between the forest and urban (control) settings in terms of the restorative effect (n = 46).

			POMS						PANAS		ROS	SVS
			*T-A*	*D-D*	*A-H*	*V*	*F*	*C*	*Negative*	*Positive*		
Before	Ave.(S.D.)	Forest	43.15 (8.03)	44.00 (5.61)	41.89 (7.46)	42.78 (10.39)	43.83 (9.31)	44.13 (8.37)	11.96 (6.22)	22.00 (9.61)	4.36 (0.95)	12.37 (4.76)
		Urban (Control)	42.39 (8.22)	43.52 (6.37)	40.33 (6.43)	41.41 (9.28)	44.87 (9.47)	43.87 (7.04)	14.20 (8.44)	20.59 (9.61)	4.19 (1.07)	11.54 (4.29)
	*p value*		0.451	0.449	0.161	0.322	0.410	0.780	0.079	0.282	0.345	0.237
	Significance		-	-	-	-	-	-	-	-	-	-
	*effect size: r*		0.113	0.114	0.208	0.148	0.124	0.042	0.259	0.161	0.141	0.176
	*statistical power: β*		0.115	0.117	0.285	0.165	0.128	0.059	0.406	0.186	0.149	0.218
After	Ave.(S.D.)	Forest	39.15 (5.97)	42.37 (4.97)	39.63 (4.51)	45.15 (9.61)	42.7 (9.25)	40.93 (5.84)	11.76 (6.11)	23.93 (9.98)	4.93 (1.09)	13.22 (4.75)
		Urban (Control)	43.98 (7.58)	43.63 (5.52)	40.96 (4.83)	36.35 (8.62)	49.54 (10.22)	45.61 (7.86)	16.26 (8.32)	21.39 (10.29)	3.52 (1.49)	9.74 (5.47)
	*p* value		0.000	0.059	0.038	0.000	0.000	0.000	0.001	0.047	0.000	0.001
	Significance		**	-	-	**	**	**	*	-	**	*
	*effect size: r*		0.586	0.278	0.304	0.729	0.591	0.519	0.460	0.291	0.576	0.469
	*statistical power: β*		0.997	0.475	0.556	1.000	0.998	0.979	0.925	0.513	0.996	0.936

Ave. = Average; S.D. = Standard Deviation; POMS = Profile of Mood States; PANAS = Positive and Negative Affect Schedule; ROS = Restorative Outcame Scale; SVS = Subjective Vitality Scale. Paired t-test (After applying Bonferroni’s correction): ** *p* < 0.001 * *p* < 0.005 - *p* > 0.005. Takayama et al. (2014) [41] was referred to and cited to arrange this table.

**Table 7:** Results of comparison between the before and after staying in terms of restorative effect (n = 46).

			POMS						PANAS		ROS	SVS
			*T-A*	*D-D*	*A-H*	*V*	*F*	*C*	*Negative*	*Positive*		
Forest	Ave.(S.D.)	Before	43.15 (8.03)	44 (5.61)	41.89 (7.46)	42.78 (10.39)	43.83 (9.31)	44.13 (8.37)	11.96 (6.22)	22 (9.55)	4.36 (0.95)	12.37 (4.76)
		After	39.15 (5.97)	42.37 (4.97)	39.63 (4.51)	45.15 (9.61)	42.70 (9.25)	40.93 (5.84)	11.76 (6.11)	23.93 (9.98)	4.93 (1.09)	13.22 (4.75)
	*p value*		0.001	0.006	0.026	0.115	0.322	0.009	0.199	0.851	0.414	0.000
	Significance		*	-	-	-	-	-	-	-	-	**
	*effect size: r*		0.485	0.395	0.324	0.233	0.148	0.378	0.191	0.029	0.122	0.58
	*statistical power: β*		0.953	0.804	0.613	0.349	0.165	0.764	0.054	0.507	0.930	0.269
Urban(Control)	Ave.(S.D.)	Before	42.39 (8.22)	43.52 (6.37)	40.33 (6.43)	41.41 (9.28)	44.87 (9.47)	43.87 (7.04)	14.20 (8.44)	20.59 (9.61)	4.19 (1.07)	11.54 (4.29)
		After	43.98 (7.58)	43.63 (5.52)	40.96 (4.83)	36.35 (8.62)	49.54 (10.22)	45.61 (7.86)	16.26 (8.32)	21.39 (10.29)	3.52 (1.49)	9.74 (5.47)
	*p* value		0.001	0.025	0.858	0.049	0.091	0.57	0.001	0.002	0.718	0.002
	Significance		*	-	-	-	-	-	*	*	-	*
	*effect size: r*		0.295	0.205	0.216	0.589	0.504	0.316	0.291	0.080	0.430	0.426
	*statistical power: β*		0.494	0.275	0.297	0.969	0.900	0.545	0.468	0.081	0.796	0.796

Ave. = Average; S.D. = Standard Deviation. Paired t-test (after applying Bonferroni’s correction): ** *p* < 0.001, * *p* < 0.005, - *p* > 0.005. Takayama et al. (2014) [41] was referred to and cited to arrange this table.

**Table 8:** Results from correlation analyses in the forest setting (n = 46).

		POMS						PANAS		ROS	SVS
		*T−A*	*D−D*	*A−H*	*V*	*F*	*C*	*Positive*	*Negative*		
**Health and Life habit**	*Health*	0.031	−0.023	−0.068	0.168	−0.051	−0.046	−0.122	0.202	0.216	−0.168
	*Exercise*	−0.026	0.054	0.080	0.066	0.158	0.085	−0.071	0.093	0.175	−0.097
	*Meal*	−0.283	−0.194	0.217	0.239	−0.182	−0.295 *	−0.107	−0.023	0.141	−0.009
	*Rest*	−0.107	−0.113	0.153	0.274	−0.122	−0.213	−0.142	0.006	0.067	−0.110
	*Life habit*	−0.186	−0.117	0.196	0.255	−0.093	−0.200	−0.133	0.025	0.156	−0.085
**Stress coping**	*Planful*	0.032	0.093	0.003	0.041	−0.074	−0.061	−0.069	−0.068	0.013	−0.210
	*Confront*	0.143	0.106	0.063	−0.274	−0.004	0.114	0.100	0.005	−0.130	−0.181
	*Seeking social support*	0.053	0.191	0.179	0.082	0.149	0.024	−0.052	0.116	0.036	−0.201
	*Accepting responsibility*	0.253	0.145	−0.017	−0.024	−0.126	−0.067	0.095	0.106	−0.008	−0.181
	*Self−control*	−0.078	−0.030	−0.135	−0.010	−0.138	−0.147	−0.105	0.168	0.232	−0.271
	*Escape*	0.086	0.140	−0.003	−0.221	0.109	0.059	−0.012	0.191	−0.023	−0.223
	*Distancing*	0.062	0.188	−0.070	−0.338 *	0.069	0.223	0.032	−0.028	−0.210	−0.339 *
	*Positive reappraisal*	0.053	0.137	−0.093	−0.019	−0.224	−0.041	−0.132	0.143	0.092	−0.075
	*Problem−focused*	0.081	0.142	−0.005	0.006	−0.128	−0.080	−0.045	0.059	0.045	−0.234
	*Emotion−focused*	0.117	0.186	−0.028	−0.260	0.046	0.124	0.001	0.142	−0.039	−0.303 *
**Resilience**	*Social support*	−0.072	0.059	−0.097	0.072	−0.054	−0.130	−0.084	0.118	0.069	−0.166
	*Self−efficacy*	−0.056	−0.077	0.033	0.082	−0.207	−0.175	−0.153	0.064	0.044	−0.201
	*Sociality*	−0.006	−0.128	−0.066	0.022	0.123	−0.097	−0.246	−0.095	−0.030	−0.272
	*Total*	−0.066	−0.038	−0.054	0.082	−0.091	−0.175	−0.180	0.067	0.049	−0.252
	*Active(extrinsic)–Active(intrinsic)*	−0.015	0.103	−0.111	−0.045	−0.183	−0.077	0.037	−0.138	−0.160	−0.147
	*Passive(extrinsic) –Active(intrinsic)*	0.044	−0.042	−0.042	−0.062	0.205	0.245	−0.055	0.105	0.077	0.015
	*Active(extrinsic) –Passive(intrinsic)*	−0.050	−0.048	0.250	0.170	0.022	−0.078	−0.112	−0.067	0.157	0.039
	*Passive(extrinsic) –Passive(intrinsic)*	0.025	−0.065	−0.041	−0.038	0.022	−0.093	0.133	0.176	−0.005	0.184
**Quality of life (QOL)**	*Physical health*	0.024	−0.069	0.069	0.230	0.008	−0.028	−0.195	0.146	0.208	−0.201
	*Psychological health*	0.067	0.035	−0.071	−0.041	−0.015	0.003	0.069	0.012	0.159	−0.365 *
	*Social relationships*	0.068	0.029	−0.054	0.055	−0.014	−0.111	0.039	0.056	0.206	−0.286
	*Environment*	−0.228	−0.182	0.007	0.195	−0.334 *	−0.335 *	−0.115	0.073	0.207	−0.080
	*Total*	−0.083	−0.024	0.084	0.148	0.026	−0.107	−0.143	0.034	0.138	−0.221

Test for no correlation (Pearson’s correlation): * *p* < 0.05.

**Table 9:** Results from correlation analyses in the urban (control) setting (n = 46).

		POMS						PANAS		ROS	SVS
		*T−A*	*D−D*	*A−H*	*V*	*F*	*C*	*Positive*	*Negative*		
**Health and Life habit**	*Health*	0.138	0.031	0.002	−0.068	−0.110	−0.095	0.210	0.089	−0.129	0.059
	*Exercise*	0.124	0.091	0.148	−0.122	0.183	−0.007	0.080	0.161	−0.190	−0.202
	*Meal*	−0.046	0.142	−0.084	−0.086	−0.338 *	0.129	−0.002	0.087	0.067	0.446 **
	*Rest*	0.051	0.117	−0.028	−0.026	−0.187	0.075	−0.118	0.212	−0.028	0.249
	*Life habit*	0.036	0.147	−0.002	−0.091	−0.181	0.090	−0.028	0.188	−0.045	0.253
**Stress coping**	*Planful*	0.037	0.047	−0.074	−0.168	−0.327 *	−0.136	0.013	0.048	−0.070	0.266
	*Confront*	0.119	0.063	−0.084	−0.237	−0.073	−0.149	0.011	0.090	−0.231	−0.016
	*Seeking social support*	0.275	0.383 **	0.263	−0.085	0.203	0.094	−0.092	0.068	−0.069	−0.160
	*Accepting responsibility*	0.216	0.235	0.197	−0.152	−0.120	0.003	−0.011	0.238	−0.172	0.131
	*Self−control*	0.051	0.087	−0.043	−0.346 *	−0.080	−0.251	0.299 *	0.143	−0.444 **	−0.072
	*Escape*	0.222	0.245	0.013	−0.365 *	0.128	−0.115	0.263	0.076	−0.401 **	−0.211
	*Distancing*	−0.001	0.011	−0.034	−0.445 **	−0.093	−0.175	0.285	0.003	−0.171	−0.010
	*Positive reappraisal*	0.189	0.135	0.029	−0.155	−0.200	−0.072	0.010	0.017	−0.247	0.170
	*Problem−focused*	0.186	0.204	0.061	−0.201	−0.184	−0.058	−0.012	0.127	−0.194	0.162
	*Emotion−focused*	0.168	0.180	0.032	−0.441 **	−0.013	−0.210	0.283	0.085	−0.400 **	−0.119
**Resilience**	*Social support*	0.241	0.221	0.161	−0.015	0.256	0.177	0.140	0.268	−0.089	−0.164
	*Self−efficacy*	−0.055	0.004	−0.020	−0.025	−0.072	0.022	0.049	0.232	−0.162	0.091
	*Sociality*	0.169	0.073	−0.019	−0.056	0.047	0.003	0.083	0.215	−0.202	−0.049
	*Total*	0.147	0.138	0.071	−0.035	0.115	0.104	0.119	0.305 *	−0.175	−0.058
	*Active(extrinsic) –Active(intrinsic)*	0.008	−0.001	0.006	0.239	−0.068	0.066	−0.143	0.165	0.106	0.115
	*Passive(extrinsic) –Active(intrinsic)*	−0.059	−0.090	−0.065	−0.179	0.044	−0.181	−0.028	−0.362 *	−0.023	−0.162
	*Active(extrinsic) –Passive(intrinsic)*	−0.009	0.097	0.049	−0.278	0.048	−0.045	0.302 *	0.245	−0.300 *	−0.080
	*Passive(extrinsic) –Passive(intrinsic)*	0.071	0.008	0.018	0.137	0.005	0.169	−0.064	−0.095	0.189	0.102
**Quality of life (QOL)**	*Physical health*	0.063	0.060	0.060	0.008	−0.073	−0.088	−0.018	0.136	−0.117	0.023
	*Psychological health*	−0.156	−0.040	0.018	0.070	0.017	−0.163	0.276	0.177	−0.191	−0.122
	*Social relationships*	0.005	0.056	−0.027	0.128	0.043	−0.062	0.001	0.287	−0.250	−0.085
	*Environment*	−0.109	0.021	−0.099	−0.072	−0.178	−0.140	0.058	0.310 *	−0.129	0.103
	*Total*	−0.129	0.025	0.161	0.090	−0.011	−0.025	0.093	0.203	0.068	−0.004

Test for no correlation (Pearson’s correlation): ** *p* < 0.01 * *p* < 0.05.

**Table 10:** Results from multiple regression analysis (step-wise, forward selection; n = 46).

		Forest setting							Urban (Control) setting									
		POMS						SVS	POMS						PANAS		ROS	SVS
		*T-A*	*D-D*	*A-H*	*V*	*F*	*C*		*T-A*	*D-D*	*A-H*	*V*	*F*	*C*	*Positive*	*Negative*		
	*R^2^*				0.114	0.197	0.112	0.219		0.275	0.114		0.131	0.27		0.147	0.294	0.295
	*adjR^2^*				0.094	0.159	0.092	0.182		0.241	0.094		0.111	0.218		0.127	0.261	0.262
	*Statistical power:β*				0.503	0.597	0.495	0.660		0.787	0.503		0.571	0.659		0.628	0.820	0.822
**Health and Life habit**	*Health*																	
	*Exercise*																	
	*Meal*												−0.338 *					
	*Rest*																	
	*Life habit*																	
**Stress coping**	*Planful*																	
	*Confront*																	
	*Seeking socialsupport*									0.383 **								
	*Accepting responsibility*																	
	*Self-control*															0.597 **	−0.452 **	
	*Escape*																	−0.66 **
	*Distancing*				−0.338 *			−0.295 *				−0.468 **						0.365 *
	*Positive reappraisal*																	
	*Problem-focused*															−0.413 *		
	*Emotion-focused*																	
**Resilience**	*Social support*																	
	*Self-efficacy*																	
	*Sociality*																	
	*Total*																	
	*Active(extrinsic)-Active(intrinsic)*											0.278 *						
	*Passive(extrinsic)-Active(intrinsic)*														−0.362 *			
	*Active(extrinsic)-Passive(intrinsic)*															0.285 *	−0.312 *	
	*Passive(extrinsic)-Passive(intrinsic)*																	
**Quality of life (QOL)**	*Physical health*																	
	*Psychological health*							-0.325 *										
	*Social relationships*																	
	*Environment*					-0.560 **	-0.335 *											
	*Total*																	

(1) The numbers are the partial regression coefficient selected as a result of the step-wise method (forward selection). (2) Shaded squares indicate items that were significant in the correlation analysis (Table 8 and Table 9). (3) PANAS’s and ROS’s that had non-significant relationships by the analysis in the forest setting were omitted from the table; ** *p* < 0.01, * *p* < 0.05.
